# Potential roles of microRNAs and ROS in colorectal cancer: diagnostic biomarkers and therapeutic targets

**DOI:** 10.18632/oncotarget.14461

**Published:** 2017-01-03

**Authors:** Jingmei Lin, Chia-Chen Chuang, Li Zuo

**Affiliations:** ^1^ Department of Pathology and Laboratory Medicine, Indiana University School of Medicine, Indianapolis, IN, USA; ^2^ Radiologic Sciences and Respiratory Therapy Division, School of Health and Rehabilitation Sciences, The Ohio State University College of Medicine, Columbus, OH, USA; ^3^ Interdisciplinary Biophysics Graduate Program, The Ohio State University, Columbus, OH, USA

**Keywords:** colorectal cancer, free radicals, microRNA, ROS, therapeutic targets

## Abstract

As one of the most commonly diagnosed cancers worldwide, colorectal adenocarcinoma often occurs sporadically in individuals aged 50 or above and there is an increase among younger patients under 50. Routine screenings are recommended for this age group to improve early detection. The multifactorial etiology of colorectal cancer consists of both genetic and epigenetic factors. Recently, studies have shown that the development and progression of colorectal cancer can be attributed to aberrant expression of microRNA. Reactive oxygen species (ROS) that play a key role in cancer cell survival, can also lead to carcinogenesis and cancer exacerbations. Given the rapid accumulating knowledge in the field, an updated review regarding microRNA and ROS in colorectal cancer is necessary. An extensive literature search has been conducted in PubMed/Medline databases to review the roles of microRNAs and ROS in colorectal cancer. Unique microRNA expression in tumor tissue, peripheral blood, and fecal samples from patients with colorectal cancer is outlined. Therapeutic approaches focusing on microRNA and ROS in colorectal cancer treatment is also delineated. This review aims to summarize the newest knowledge on the pathogenesis of colorectal cancer in the hopes of discovering novel diagnostic biomarkers and therapeutic techniques.

## INTRODUCTION

Over one million new cases of colorectal cancer are identified annually, making it the third most common cancer worldwide [1]. It is largely diagnosed in individuals older than 50 years of age. Thus, standard colonoscopy is recommended starting at the age of 50 to improve early screening and detection [2]. The multifactorial etiology of colorectal cancer consists of both genetic and epigenetic factors. The hereditary syndromes comprise familial adenomatous polyposis, Lynch syndrome (hereditary nonpolyposis colon cancer), MYH-associated polyposis, and juvenile polyposis. Inflammatory bowel disease is also considered a risk factor [3, 4]. Moreover, inappropriate diets and lifestyles are known to associate with increased colorectal cancer incidence. Some risk factors include high intake of red meat/processed food, heavy alcohol consumption, obesity, smoking, and physical inactivity. It is suggested that the development of colorectal cancer can be prevented by regular exercise and proper diets [5, 6]. The progression of normal colonic mucosa to invasive colorectal cancer requires multiple steps of molecular alterations. The estimated time interval of malignant transformation from normal mucosa to adenomatous polyp and invasive adenocarcinoma is 5-10 years [7]. Five year survival rates for Tumor Node Metastasis (TNM) stages I to IV colorectal cancer are 90%, 80%, 60%, and 8%, respectively [8].

MicroRNA is encoded within the genomes of a variety of eukaryotes, including over 2,500 human mature microRNA sequences in the miRBase database [9–11]. MicroRNAs are evolutionarily conserved, single-stranded noncoding RNA molecules of 19-24 nucleotides, which can suppress gene expression at posttranscriptional levels. MicroRNAs concurrently modulate the expression levels of dozens or more messenger RNA (mRNA), and any given mRNA sequence may be targeted by several different microRNAs [9, 10, 12]. To date, microRNAs have been predicted to target and control the expression of at least 30% of all protein-coding genes, and they participate the regulation of nearly every cellular process studied so far [13]. Specifically, microRNAs appear to be involved in multiple pathophysiological networks and in the pathogenesis of a broad-spectrum of human diseases, including cancer and inflammation [14–21]. The greater stability of microRNAs relative to mRNAs supports the use and development of microRNAs as promising targets in diagnostic and therapeutic applications of various diseases [22]. Indeed, plasma microRNAs have been used for early detection of cancer such as colorectal cancer, which is essential in improving prognosis [23, 24]. MicroRNAs also demonstrate high sensitivity and specificity in cancer diagnosis, further confirming their potential as biomarkers [24].

Over the past 15 years, researchers have identified distinct aberrant microRNA expression profiles in tumor tissue, peripheral blood, and fecal samples of colorectal cancer patients, suggesting the critical roles that microRNAs play in the pathogenesis of oncogenic transformation. In addition, reactive oxygen species (ROS), serving as important cell signaling molecules, are involved in the progression of cancer cells as well as in microRNA expression. Elevated ROS levels and accumulated mutations due to oxidative DNA damage are prominent in cancer cells, favoring the survival and growth of cancer [25, 26]. Particularly in colorectal cancer, the irritated intestines and altered gut microbiota composition can contribute to additional production of intestinal ROS. Indeed, a marked increase in oxidative stress markers such as 8-oxodG (an indicator of DNA oxidation) was observed in colorectal cancer patients, suggesting the potential role of ROS in colorectal cancer [27]. Growing evidence from cancer studies has revealed that microRNA expression alters in response to ROS exposure [28]. In addition to the ROS-mediated tumor progression, it is likely that ROS are also involved in the microRNA-related mechanisms of promoting colorectal carcinogenesis. Understanding the interplay between microRNAs and ROS is paramount since both have been shown to be dysregulated in cancers. Herein, the review focuses on the current understanding of microRNAs and ROS in the pathogenesis and potential diagnostic and therapeutic implication in colorectal cancer.

## ABERRANT MICRORNA PROFILES AND ROS LEVELS IN COLORECTAL CANCER

### MicroRNA profiles in colorectal tissues, plasma, and stool samples

Nearly 400 dysregulated microRNAs have been identified in colorectal cancer in the past decade, yet minimal consistency of microRNA expression profile is reported. Despite the striking potential of microRNAs as biomarkers of cancer, the transition of microRNAs from bench to clinical use remains challenging as the detection techniques including the commonly used qRT-PCR are needed to be optimized. For example, the selection of suitable reference genes for data normalization in the qRT-PCR analysis is highly subjective, which may lead to inconsistency among different studies. The intrinsic properties of microRNAs, such as a high degree of sequence similarity within the same family, tissue-specific expression, and small-quantity, also cause certain limitations of these detection methods [24]. In addition, by far a considerable amount of results in the literature is derived from retrospective cohorts, thereby limiting the prognostic significance of microRNAs and possibly contributing to some inconsistent results.

As listed in Table 1, aberrantly elevated microRNAs have been frequently found in cancerous colon tissues. Overexpressed miR-31 is commonly observed in colorectal tumor tissues, and is associated with tumor prognosis [29]. Given the variable and diverse anatomic locations of colorectal cancer, treatment management, tissue processing, cohorts of normal control, and analytical methods, which all might impact results, it is not surprising that the findings are not consistent. Similarly, there is no established general consensus on the normalization of circulating microRNAs despite the distinct microRNA expression profiles observed in sera or plasma of patients with colorectal cancer (Table 2). Among these highly expressed circulating microRNAs, several are found in the peripheral blood mononuclear cells and others are known to be secreted by the tumor tissues. Indeed, miR-21 is abundantly present in colorectal tumor tissues and is secreted to the circulation. Levels of miR-21 in the serum sample decrease after surgical removal of primary tumor, suggesting the need of establishing an in-depth evaluation of circulating microRNAs for diagnosis [30]. Fecal occult blood testing is a useful option for early detection, but the sensitivity is low [31]. So far, less than 40% of colorectal cancers are detected in the early stage [32]. Therefore, there is a strong demand for the development of accurate and noninvasive markers. Given the continuous releasing of colonic epithelia into the lumen, detecting microRNA in fecal samples from colorectal cancer patients is a promising tool for the early diagnosis of colorectal cancer. As listed in Table 3, altered microRNA expression profiles are found in stool samples from patients with colorectal cancer. Among them, miR-21, miR-106a, miR-143, and miR-145, have been found by at least two independent groups.

**Table 1:** Dysregulated microRNAs in tumor tissues in patients with colorectal cancer

**Dysregulation**	**Number of studies**	**microRNAs and references**
Upregulated	17	miR-21[44, 45, 64, 65, 71, 75–79, 82, 84, 116–120]
	12	miR-31[45, 64–71, 77, 78, 116]
	9	miR-135b[45, 65, 70, 71, 77, 83, 93, 116, 121]
	8	miR-20a[66, 71, 75, 76, 78, 79, 94, 95]; miR-183[45, 66, 67, 70, 71, 77, 78, 116]
	7	miR-18a[66, 70, 78, 95, 121–123]
	6	miR-19a[71, 77, 78, 95, 116, 121]; miR-96[65, 70, 77, 78, 93, 116]; miR-181b[76, 78, 79, 117, 124, 125]
	5	miR-92[66, 67, 69, 77, 94]; miR-106a[75–79] ; miR-203[67, 76–79]
	4	miR-17[67, 71, 95, 122]; miR-17-5p[66, 75, 77, 78]; miR-19b[71, 78, 95]; miR-20[67, 77, 116, 126]; miR-25[67, 77, 78, 122]; miR-182[70, 71, 77, 78]; miR-200c[69, 77, 124, 125]; miR-224[70, 77, 78, 121]
	3	miR-29a[77, 78, 120]; miR-93[67, 71, 78]; miR-106b[78, 121, 122]; miR-130b[71, 77, 78]; miR-142-3p[69, 71, 77]; miR-191[75, 77, 125]; miR-221[71, 75, 127];
	2	miR-15a[71, 77]; miR-15b[77, 125]; miR-17-3p[70, 77]; miR-29b[78, 120]; miR-32[70, 75]; miR-34a[77, 78]; miR-92a[44, 122]; miR-95[77, 78]; miR-98[71, 77];miR-105[77, 93]; miR-107[75, 77]; miR-135a[67, 77]; miR-148a[71, 77]; miR-182*[70, 77];miR-188[69, 70]; miR-200a*[77, 94]; miR-210[69, 77]; miR-223[67, 75];miR-301b[45, 121]; miR-320[77, 94]; miR-324-5p[71, 77]; miR-424[121]; miR-493[45, 93]; miR-513a-5p[82, 120]; miR-552[70, 93]; miR-584[70, 93]; miR-let-7g[77, 124]
	1	miR-1[120]; miR-7[93]; miR-10a[77]; miR-19b-1[95]; miR-24-1[75]; miR-27a[77]; miR-29b-2[75]; miR-30b[120]; miR-30c[75]; miR-33[70]; miR-92a-1[95]; miR-103[77]; miR-122a[77]; miR-128a[77]; miR-128b[75]; miR-133b[67]; miR-134[77]; miR-135b*[83]; miR-141[77]; miR-142-5p[77]; miR-145[120]; miR-146[77]; miR-147[77]; miR-150[75]; miR-151[77]; miR-154*[77]; miR-155[75]; miR-181a[77]; miR-181c[77]; miR-183*[83]; miR-186[77]; miR-191*[94]; miR-194[77]; miR-197[77]; miR-199a-3p[120]; miR-200a[69]; miR-200b[77]; miR-213[77]; miR-214[71]; miR-215[77]; miR-216[77]; miR-219[77]; miR-222[77]; miR-296-3p[93]; miR-301[77]; miR-302a[94]; miR-330[77]; miR-338[77]; miR-338-3p[120]; miR-339[77]; miR-362[45]; miR-370[77]; miR-373[77]; miR-374[77]; miR-382[45]; miR-432*[94]; miR-451[120]; miR-483-3p[93]; miR-492[94]; miR-494[82]; miR-500[82]; miR-503[70]; miR-510[94]; miR-512-5p[94]; miR-513[94]; miR-513b[82]; miR-513c[82]; miR-526c[94]; miR-527[94]; miR-542-5p[70]; miR-549[93]; miR-582-5p[71]; miR-592[93]; miR-622[128]; miR-708[45]; miR-766[83]; miR-886[45]; miR-892b[82]; miR-938[128]; miR-1238[128]; miR-1247[93]; miR-1260[120]; miR-1269[93]; miR-1290[128]; miR-1827[93]; miR-3144-3p[93]; miR-3180-3p[93]; miR-4326[93]; miR-HS-29[70]; miR-HS-287[70]; miR-let-7f[71]
Downregulated	14	miR-145[45, 64, 66–69, 77, 78, 82, 83, 93, 94, 116, 129]
	10	miR-143[64, 66, 67, 69, 78, 82–84, 129, 130]
	8	miR-1[45, 70, 71, 78, 82, 93, 128, 131]
	7	miR-195[77, 78, 82, 83, 93, 105, 132]
	6	miR-378[70, 78, 82, 83, 93, 121]
	5	miR-133a[45, 70, 71, 78, 82]; miR-133b[45, 82, 83, 116, 128]; miR-139-5p[45, 71, 82, 83, 93]; miR-192[67, 69, 82, 83, 133]; miR-215[67, 71, 82, 83, 133]
	4	miR-30a-3p[70, 77, 78, 116]; miR-375[70, 71, 83, 134]; miR-422a[71, 78, 83, 93]
	3	miR-9[45, 70, 72]; miR-10b[70, 78, 82]; miR-16[67, 83, 135]; miR-26b[67, 83, 94]; miR-30b[82, 83, 94]; miR-30c[78, 82, 83]; miR-138[45, 83, 128]; miR-139[70, 77, 78]; miR-194[82, 83, 133]; miR-363[70, 82, 93]; miR-378*[82, 83, 121]; miR-490-3p[82, 93, 128]; miR-497[70, 78, 82]; miR-let-7a[67, 136, 137]
	2	miR-9*[70, 82]; miR-28-3p[71, 120]; miR-30a*[82, 83]; miR-30a-5p[70, 78]; miR-30e[82, 83]; miR-101[83, 94]; miR-125b[69, 77]; miR-137[70, 82]; miR-149[71, 77]; miR-150[120, 138]; miR-192*[82, 83]; miR-204[45, 71]; miR-320a[139]; miR-328[70, 116]; miR-365[82, 140]; miR-486-5p[83, 93]; miR-551b[70, 93]; miR-598[82, 83]; miR-642[70, 71];
	1	miR-1[120]; miR-7-1*[82]; miR-20b[70]; miR-22[141]; miR-23b[120]; miR-24-1*[82]; miR-26a[83]; miR-27b[82]; miR-28-5p[82]; miR-30a[82]; miR-30e*[83]; miR-31[82]; miR-31*[82]; miR-34c[142]; miR-34a[142]; miR-99a[83]; miR-100[83]; miR-113; miR-122[82]; miR-124a[116]; miR-125a[78]; miR-126[143]; miR-127-3p[83]; miR-129[116]; miR-133[117]; miR-139-3p[120]; miR-140-5p[83]; miR-143*[82]; miR-144[82]; miR-144*[83]; miR-147[70]; miR-186[83]; miR-190[83]; miR-191[67]; miR-193b[69]; miR-196a[67]; miR-200b[83]; miR-203[118]; miR-212[69]; miR-214[69]; miR-218[82]; miR-299-5p[71]; miR-342-3p[83]; miR-345[144]; miR-362-3p[82]; miR-378c[93]; miR-381[145]; miR-383[93]; miR-411[83]; miR-422b[78]; miR-424[120]; miR-451[83]; miR-455[94]; miR-484[94]; miR-485-3p[71]; miR-486[70]; miR-506[146]; miR-511[70]; miR-582-5p[82]; miR-590-5p[82]; miR-622[147]; miR-628-3p[93]; miR-628-5p[93]; miR-636[83]; miR-650[70]; miR-885-5p[45]; miR-886-3p[71]; miR-892b[120]; miR-1288[120]; miR-1297[93]; miR-1305[120]; miR-3151[93]; miR-3163[93]; miR-3622a-5p[93]; miR-3656[93]

**Table 2:** Dysregulated microRNAs in sera or plasma samples in patients with colorectal cancer

**Source**	**Dysregulation**	**Number of studies**	**microRNAs and references**
Sera	Upregulated	3	miR-92a[85, 148, 149]; miR-221[149–151]
		2	miR-21[45, 152]; miR-210[85, 149]
		1	miR-19a[149]; miR-22*[149]; miR-24[149]; miR-92[153]; miR-125a-5p[149]; miR-134[150]; miR-141[154]; miR-146a[150]; miR-320a[85]; miR-376a[149]; miR-378[85]; miR-423-5p[85]; let-7e[149] ; miR-222[150]; miR-423-5p[85]
	Downregulated	2	miR-143[85, 89]
		1	miR-10a[149]; miR-103[85]; miR-106a[85], miR-107[85]; miR-141[149]; miR-145[96]; miR-150[149]; miR-151-5p[85]; miR-188-3p[149]; miR-192[149]; miR-199a-3p[85]; miR-224*[149]; miR-382[85]; miR-425*[149]; miR-495[149]; miR-572[149]; miR-601[149]; miR-720[149]; miR-760[149]; let-7a[149]; let-7d[85]

**Table 3:** Dysregulated microRNAs in fecal samples in patients with colorectal cancer

**Source**	**Dysregulation**	**Number of studies**	**microRNAs and references**
Feces	Upregulated	4	miR-21[44, 86, 155, 156]
		2	miR-106a[155, 157]
		1	miR-18a[156]; miR-19a[156]; miR-20a[86]; miR-92[86]; miR-92a[44]; miR-96[86]; miR-106a[86]; miR-135a[156]; miR-135b[156]; miR-144[158]; miR-203[86]; miR-326[86]
	Downregulated	2	miR-143[86, 87]; miR-145[86, 87]
		1	miR-16[86]; miR-125b[86]; miR-126[86]; miR-320[86]; miR-484-5p[86]

### ROS in colorectal cancer

ROS, including superoxide (O_2_^·-^), hydroxyl radical (^·^OH) and hydrogen peroxide (H_2_O_2_), are generated under physiological conditions and serve as important mediators in multiple cell signaling pathways. Despite their importance, excessive ROS can oxidize major cellular components (e.g., DNA, lipids, and proteins), resulting in irreversible damages [33]. Normally, the cellular levels of ROS are carefully monitored by the body's natural antioxidant defense system in order to maintain redox homeostasis. When such homeostasis is disrupted (termed oxidative stress), either due to ROS overproduction or compromised antioxidant function, it can give rise to pathological conditions that ultimately leads to diseases [34, 35]. Compared to normal cells, the basal level of ROS has been shown to elevate in cancer cells, which is mainly attributed to increased metabolic activity and altered cellular signaling [26]. Originating from the epithelium in the intestine, colorectal cancer cells have a high metabolic rate and often divide rapidly, potentially causing DNA oxidation [27]. These ROS-induced genetic mutations as well as transcription factor modulations (e.g., hypoxia inducible factor-1) are crucial in the regulation of gene expression relative to cancer cell survival, growth, invasion, and metastasis, contributing to all three stages of carcinogenesis (initiation, promotion and progression) [25, 27]. Cancer cells are normally accompanied with strong antioxidant defenses, generating a powerful ROS scavenging capacity that can adapt to a highly oxidized environment and avoid apoptosis [25, 26]. Sustained and excessive ROS promote oncogenic activity and genomic instability, contributing to carcinogenesis [28]. The association between colorectal cancer and oxidative stress has been identified in the past decades. Additionally, the increased levels of oxidative stress biomarkers, such as 8-oxodG in DNA, suggest that the ROS are markedly elevated in the whole blood of patients with colorectal cancer [27].

In a study of primary rat colonocytes, Oberreuther-Moschner *et al*. observed that cells from the lower aspect of colon crypts, mostly proliferating cells, are more sensitive to ROS-H_2_O_2_ damage [36]. The stem/progenitor cells’ capacities of self-renewal and differentiation are also largely influenced by varied redox environments. Therefore, these cells are putative targets for colon cancer treatment [27]. Recent efforts have focused on the development of effective therapies that combat cancer cells, by facilitating the induction of apoptosis *via* drug-induced ROS. These methods include various chemotherapeutic/anti-cancer drugs [25]. In contrast, lowering oxidative stress or increasing the total antioxidant capacity through a vegetable- and fruit-rich diet has shown to decrease the potential risk of colorectal cancer. Western diets that commonly consist of red meat, which contains high quantity of iron, are not favorable since heme iron promotes cell transformation and oxidative DNA damage by exacerbating oxidative stress in the body [25]. Acting as a double-edge sword, intracellular ROS levels play critical roles in determination of the fate for cancer cells. As such, the survival of cancer cells in the presence of either very high or low ROS levels is not favorable, and therapies targeting redox disruption should be carried out with cautions [33].

### Interaction of microRNAs and ROS

Since the dysregulation of microRNAs and ROS are both observed in colorectal cancer, it is essential to understand their potential role of interaction in relation to colorectal carcinogenesis and progression. Among those microRNAs presented in our tables, miR-210 overexpression has been shown to increase ROS production in colorectal cancer cells [37, 38]. The augmented ROS can be attributed to compromised mitochondrial activity as miR-210 inhibits mitochondrial iron-sulfur cluster scaffold homologue and the assembly iron-sulfur cluster [37, 39]. MiR-210 also induces ROS generation under hypoxic condition, leading to a poor prognosis in colorectal cancer [37, 38]. Furthermore, Tagscherer *et al*. observed miR-210-induced colorectal cancer apoptosis. However, the roles of elevated ROS and miR-210 levels in the regulation of apoptosis and their biological relevance in colorectal cancer have not be thoroughly elucidated and require further investigation [37]. Additionally, overexpressed miR-141 and miR-200a can modulate oxidative stress by targeting p38a and potentiate tumor growth [28].

Accumulating studies reveal that ROS can alter the expression of several microRNAs. For example, exogenous H_2_O_2_ exposure has led to the upregulation of miR-21 while lowered the expressions of miR-27a*, miR-27b*, miR-29b, and miR-328 [28]. It has been shown that ROS regulate microRNA expression through microRNA biogenesis, transcription factors, and epigenetic alterations. In addition, microRNA response can be abrogated when ROS are scavenged [28]. Studies indicated that ROS upregulate miR-21 expression, actively involving in the initiation of cancer metastasis [40, 41]. MiR-21 plays an essential role in many aspects of colorectal carcinogenesis as its upregulation has been found in the tumor tissues, serum, and stool of patients with colorectal cancer (Tables 1, 2, 3). Although the targets of miR-21, including several tumor suppressors, have been successfully identified, the continuation of extensive research on this matter is essential to elucidate the diverse mechanisms of miR-21 in the cancer development [42, 43]. It is suggested that one of the potential mechanisms utilized by miR-21 to promote tumorigenesis is through the alteration of cellular ROS levels [43]. In the study of Zhang *et al*., miR-21 has been shown to suppress SOD3 directly or SOD2 indirectly by reducing TNF-α production, thereby inhibiting the dismutation of O_2_^·-^ to the less damaging molecule of H_2_O_2_ [43]. In the miR-21 overexpressing cells that are under irradiation (IR), the accumulated O_2_^·-^ may be involved in the IR-induced cell transformation. Along with other targets of miR-21, such modulations of ROS levels contribute to the carcinogenesis [43]. Although miR-21-ROS interaction has not been documented in colorectal cancer yet, further research may be necessary to explore towards this direction, and perhaps with other microRNAs, given their tight association with the colorectal cancer.

Certain microRNAs, such as miR-34a, have been shown to inhibit ROS synthesis by silencing the genes that code for mitochondrial complexes and other ROS-producing enzymes, contributing to apoptosis resistance. The restoration of these microRNAs is suggested to sensitize the tumor in response to IR-induced oxidative effects [28]. Clearly, ROS and microRNAs are capable of interacting synergistically or antagonistically to influence the complex and multiphase development of cancer. However, limited effects have been observed in the application of antioxidants or microRNAs in cancer treatment, and the functional consequences of individual microRNA in colorectal cancer patients are still largely unknown. Further exploration on the ROS-microRNAs network may provide powerful therapeutic potential for colorectal cancer as microRNAs can be utilized to enhance ROS-induced apoptosis or alleviate ROS-mediated oxidative stress [28].

## MICRORNA AS A POTENTIAL CARCINOGENIC DRIVER

The multifactorial etiology of colorectal cancer involves both genetic and epigenetic alterations of proto-oncogenes and tumor suppressor genes, which leads to complicated aspects of tumorigenesis, including cell proliferation, apoptosis, genomic stability, angiogenesis, metastasis and chemoresistance. Increasing evidence supports a specific and important role of noncoding genomic sequences, including microRNA in carcinogenesis. MicroRNAs exert various biological functions in tumorigenesis by altering the expression of oncogenes and/or tumor suppressors. MicroRNAs generally regulate gene expression by binding to the 3′ untranslated region (UTR) of their target mRNAs to repress translation [9, 10, 12]. Computational methods play an essential role in predicting proposed targets. In the past decade, multiple target genes of microRNAs in colorectal carcinogenesis have been proposed, validated, and confirmed in variable signaling pathways (Table 4). Here we focus on the microRNAs with the best evidence as drivers of carcinogenesis.

**Table 4:** Colorectal cancer-associated microRNAs and their validated gene targets

	MicroRNA	Confirmed gene target	References
Oncogene	miR-9	E-cadherin	[159]
	miR-17	RND3	[126]
	miR-18a	ATM	[160]
	miR-19a	TF	[161]
	miR-21	PDCD4, PTEN, RASA1, Rho B, TGFβR2	[54, 56–63]
	miR-26b	E3 ubiquitin ligase DIP1	[162]
	miR-30	GRP78	[163]
	miR-31	RASA1	[73]
	miR-32	PTEN	[164]
			[44, 165–169]
	miR-92a	PTEN	[170]
	miR-95	SNX1	[171, 172]
	miR-106a	RB1, TGFβR2	[80, 81]
		
	miR-135a	Metastasis suppressor 1	[173]
		
		
	miR-191	C/EBPβ	[174]
		
		
	miR-214	FGFR1	[175]
	miR-224	p21, MBD2, PHLPP1, PHLPP2, SMAD4	[176–179]
	miR-499-5p	FOXO4, PDCD4	[180]
		
	miR-675	RB	[181]
Tumor suppressor	miR-7	YY1	[182]
	miR-16	CDK6, cyclin D1, survivin	[135, 183]
	miR-22	p21	[184]
	miR-28-5p	CCND1, HOXB3	[185]
	miR-33a	Pim-1	[186]
	miR-34	Axin2, snail1	[187, 188]
	miR-34a	PDGFRα, LDHA, MDM4, SIRT1	[44, 165–167, 169]
	miR-93	CCNB1, ERBB2, Smad 7	[189, 190]
	miR-100	Lgr5	[191]
	miR-124	iASPP, STAT3	[114, 192]
	miR-126	CXCR4, phosphatidylinositol 3-kinase	[193–195]
	miR-127	BCL6	[196]
	miR-133b	TBPL1	[197]
	miR-139	RAP1B, IGF-IR	[198, 199]
	miR-139-5p	IRS1, Notch1,	[200]
	miR-143	DNMT3A, HK2, IGF-IR, MACC1	[89–92]
	miR-144	ROCK 1	[201]
	miR-145	DFF45, FLI1, IRS1, N-RAS, PAK4, p70S6K1, paxillin, STAT1, YES	[96, 98–103]
	miR-148a, b	Bcl-2, CCK2R	[202, 203]
	miR-181a miR-199a-5p	GRP78	[163]
	miR-195	Bcl-2	[105]
	miR-203	Hakai	[204]
	miR-204	TFAM	[205]
	miR-215	DTL	[206]
	miR-218-5p	BMI1	[207]
	miR-221	MBD2	[178]
	miR-320a	β-catenin, neuropilin 1	[139, 208]
	miR-339-5p	PRL-1	[209]
	miR-342	DNMT1	[210]
	miR-362-3p	E2F1, PTPN1, USF2	[211]
	miR-365	Bcl-2, cyclin D1	[140]
	miR-375	PIK3CA	[212]
	miR-381	LRH-1	[145]
	miR-455	RAF1	[213]
	miR-497	IGF-IR	[214]
	miR-506	EZH2	[146]
	miR-622	K-Ras	[147]
	miR-627	JMJD1A	[215]
	miR-1915	Bcl-2	[216]
	Let-7	K-Ras, MMP11, PBX3	[217]

Abbreviation: ARL2, ADP-ribosylation factor-like protein 2; ATM, ataxia telangiectasia mutated; BIM, BCL-2-interacting mediator of cell death; CCK2R, cholecystokinin-2 receptor; CCND1, cyclin D1; COX-2, cyclooxygenase-2 ; DAPK, death-associated protein kinase; DFF45, DNA fragmentation factor-45; DNMT1, DNA methyltransferase 1; DNMT3A, DNA methyltransferases 3A; DTL, denticleless protein homolog; FGFR1, fibroblast growth factor receptor 1; FLI1, friend leukemia virus integration 1; HIF-2α, hypoxia-inducible factor-2α; HK2, hexokinase 2; iASPP, apoptosis-stimulating protein of p53; IGF-IR, type I insulin-like growth factor receptor; IRS2, insulin receptor substrate 2; LDHA, lactase dehydrogenase A; Lgr5, leucine-rich repeat-containing G protein-coupled receptor 5; LRH-1, liver receptor homologue 1; KLF4, krüppel-like factor 4; K-Ras, Kirsten rat sarcoma; JMJD1A, Jumonji domain containing 1A; MACC1, metastasis-associated in colon cancer-1; MAPK, mitogen-activated protein kinase; NIRF, Np95 ICBP90 ring finger; PDCD4, programmed cell death 4; PHLPP, PH domain leucine-rich-repeats protein phosphatase; PI3K, phosphatidylinositol-3 kinase; PIAS3, the protein inhibitor of activated STAT3; PIK3CD, phosphoinositide 3-kinase catalytic subunit delta; PRL-1, phosphatases of regenerating liver-1; PDGFRα, platelet-derived growth factor receptor α; PPARγ, peroxisome proliferatior-activated receptor γ; PTEN, phosphatase and tensin homologue; RAF1, proto-oncogene serine/threonine-protein kinase; RASA1, RAS p21 GTPase activating protein 1; Rho B, ras homolog gene family, member B; ROCK1, Rho-associated coiled-coil containing protein kinase 1; SNX1, sorting nexin 1; TBPL1, TATA-box binding protein like 1; TF, tissue factor; TFAM, mitochondrial transcription factor A; YY1, Yin Yang 1.

### Oncogenes

#### miR-21

Oncogenic miR-21 is one of the most extensively studied microRNAs. Its expression is often upregulated in tumor tissue, sera, and stool samples from patients with colorectal cancer [44–46]. Remarkably, serum miR-21 has been shown to be a promising biomarker of colorectal cancer for early detection and prognosis [30]. The expression level of miR-21 is associated with TNM staging, recurrent-free cancer-specific survival, and overall survival [46, 47]. In addition, the expression of miR-21 is decreased after chemotherapy, which is related to tumor response [48]. Several studies have shown that miR-21 overexpression significantly increases the resistance of tumor cells to 5-fluorouracil and radiation in colon cancer cells [49]. Furthermore, the knockdown of miR-21 reversed these effects on tumor cells by increasing the sensitivity to 5-fluorouracil chemotherapy [49, 50]. One study suggests that miR-21 mediates resistance through the regulation of Sprouty 2 protein, a tumor suppressor, which enhances the cytotoxic effect of 5-fluorouracil in colon cancer cells [51]. These findings indicate that targeting miR-21 could enhance the sensitivity of cancer cells to chemoradiotherapy.

**Figure 1:**
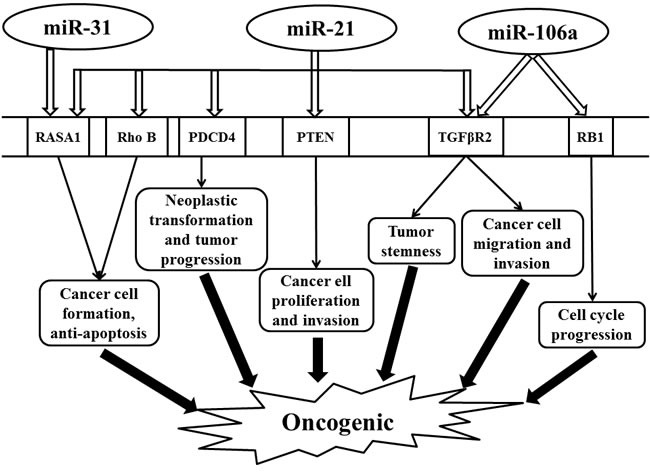
Schematic illustrating the oncogenic effect regulated by miR-31, miR-21, and miR-106a in colorectal cancer Abbreviations: PDCD4, programmed cell death 4; PTEN, phosphatase and tensin homologue; RASA1, RAS p21 GTPase activating protein 1; RB1, retinoblastoma 1; Rho B, Ras Homolog Family Member B; TGFβR2, transforming growth factor β receptor 2.

MiR-21 participates in many facets of tumorigenesis including cell proliferation, apoptosis, tumor stemness, and invasion [52–55]. Transforming growth factor β receptor 2 (TGFβR2), programmed cell death 4 (PDCD4), Rho B, PTEN, and RAS p21 GTPase activating protein 1 (RASA1) are the validated target genes by miR-21 (Figure 1) [54, 56–63]. MiR-21 overexpression is associated with induction of tumor stemness through the downregulation of TGFβR2 and the augmentation of β-catenin TCF/LEF signaling pathway [54]. As a tumor suppressor, PDCD4 inhibits tumor progression and neoplastic transformation, while miR-21 restrains the functions of PDCD4 by directly suppressing its expression [57]. MiR-21 also regulates RAS signaling pathway to affect colon cancer cell behaviors *via* direct effect on RASA1 and Rho B (Figure 1) [58, 59]. In addition, miR-21 regulates the biological behavior of human colorectal cancer cells through PTEN/PI-3 K/Akt signaling pathway [60]. Taken together, these findings suggest miR-21 as a carcinogenesis instigator and potential therapeutic target for colon cancer treatment worthy of further investigation.

#### miR-31

Uniformly elevated expression of miR-31 is also observed in tumor tissue of colorectal cancer patients [64–70]. The expression level of miR-31 is positively correlated with the tumor TNM staging [65, 71]. MiR-31 is involved in cell proliferation and apoptosis by activating RAS signaling pathway through the inhibition of its target gene, RASA1, thereby enhancing cancer cell growth (Figure 1) [72, 73]. Functional analysis has demonstrated that an inhibitor of miR-31 has an anti-tumor effect [74]. Thus, miR-31 may serve as a diagnostic biomarker and a promising therapeutic target in colon cancers.

#### miR-106a

As an oncogene, miR-106a is upregulated in colorectal cancer, which is also associated with tumor metastasis [75–80]. Retinoblastoma 1 (RB1) and TGFβR2 are validated direct target genes of miR-106a [80, 81]. RB1, an important tumor suppressor gene involved in cell cycle, is directly regulated by miR-106a [81]. In addition, miR-106a is highly expressed in metastatic colon cancer cell lines that can enhance tumor migration and invasion by modifying TGFβR2 directly (Figure 1) [80].

## TUMOR SUPPRESSOR GENES

### miR-143

As a tumor suppressor, miR-143 is downregulated in tumor tissue, sera, and fecal samples of patients with colorectal cancer [82–87]. Its expression is also decreased in the front-specific tumor invasion in liver metastasis [88]. Reduced expression of miR-143 is associated with aggressive mucinous phenotype and is strongly correlated with clinical stage and nodal metastasis [71, 89]. The augmented postchemotherapy level of miR-143 is thought to be associated with a better prognosis [48].

**Figure 2:**
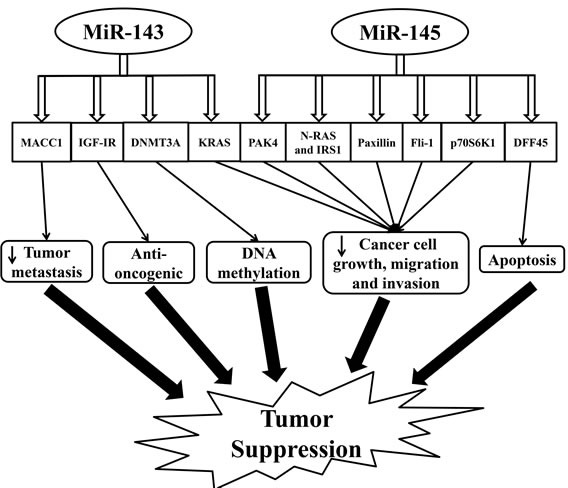
Schematic illustrating the tumor suppression effect regulated by miR-143 and miR-145 in colorectal cancer Abbreviations: DNMT3A, DNA methyltransferases 3A; DFF45, DNA fragmentation factor-45; IGF-IR, insulin-like growth factor-I receptor; IRS1, insulin receptor substrate-1; KRAS, Ki-ras2 Kirsten rat sarcoma viral oncogene homolog; MACC1, metastasis-associated in colon cancer-1; p70S6K1, phosphorylated 70-kDa ribosomal protein S6 kinase 1; PAK4, p-21 activated kinase 4.

Hexokinase 2 (HK2), metastasis-associated in colon cancer-1 (MACC1), insulin-like growth factor-I receptor (IGF-IR), KRAS, and DNA methyltransferases 3A (DNMT3A) are the confirmed target genes of miR-143 [89–92]. Studies have shown that HK2 is involved in glucose metabolism in colon cancer cells [90]. MACC1 has been identified to express highly in colorectal cancer cells and promotes tumor metastasis through activating a metastasis-inducing HGF/MET signaling pathway [91]. MiR-143 plays a role in suppressing colorectal cancer cell growth *via* directly inhibiting KRAS [69]. IGF-IR, a known oncogene, has expression levels that are inversely correlated with miR-143 expression in human tumor tissues (Figure 2). In an IGF-IR-dependent manner, miR-143 overexpression has been shown to boost colorectal cancer chemosensitivity to oxaliplatin treatment [89]. In addition, specific changes in DNA methylation patterns are associated with human cancers. Methylation changes to the genome are controlled by DNA methyltransferases (DNMT). MiR-143 specifically regulates DNMT3A that reflects its role in the regulation of DNA methylation (Figure 2) [92]. Taken together, through modulating multiple targets, miR-143 provokes potent effects on cancer cell growth and tumorigenesis.

#### miR-145

MiR-145 is a commonly studied tumor suppressor microRNA in colorectal cancer, which is downregulated in tumor tissue, sera, and fecal samples [29, 83, 87, 93–96]. Its decreased expression is even present in the front-specific tumor invasion in liver metastasis [88]. Similar to miR-143, the increased postchemotherapy level of miR-145 is predictive of a better prognosis [48].

Reduced expression of miR-145 has been observed in the non-mutated adenomatous polyposis coli, suggesting its role in the initiation of colorectal tumor development [97]. PAK4, N-RAS, IRS1, paxillin, FLI1, DFF45, and p70S6K1 are the known recognized targets of miR-145 (Figure 2) [96, 98–105]. PAK4, a subfamily of serine/threonine kinases linked to cell growth, motility, and cytoskeletal dynamics, is greatly involved in oncogenic signaling pathways [101]. Overexpressed miR-145 exerts its anti-tumor function by modulating a target gene, PAK4, which blocks the activation of AKT and ERK1/2 pathways, thus inhibiting tumor growth [101]. In addition, miR-145 also blocks the activation of AKT and ERK1/2 pathways and the expression of HIF-1 and VEGF *via* directly targeting N-RAS and IRS1, leading to the inhibition of tumor growth [96]. Meanwhile, miR-145 is involved in inhibiting cell proliferation, migration, and invasion by targeting paxillin [99]. MiR-145 also regulates the 3′-UTR of Fli-1 mRNA. The downregulation of Fli-1 has a profound effect on the growth of colon cancer [102]. DNA fragmentation factor-45, a direct target by miR-145, plays an essential role in apoptosis, a crucial aspect of tumorigenesis [106]. MiR-145 is a key regulator of intestinal cell differentiation by directly targeting SOX9, a marker of undifferentiated progenitors in the colonic crypts [100]. However, whether SOX9 is oncogenic remains controversial as inactivating mutation of this gene is frequent in colorectal cancer [107]. Indeed, Bastide *et al*. observed the presence of microadenomas in the intestinal epithelium of SOX9-knockout mice. SOX9 acts a regulator of Wnt/beta-catenin pathway and a transcriptional target to maintain intestinal epithelium homeostasis [108]. In addition, SOX9 expression is an important biomarker for the prediction of colorectal cancer relapse [109]. MiR-145 also directly targets catenin δ-1 and contributes largely to oncogenic Wnt/beta-catenin signaling in human colon cancer cells (Figure 2) [104]. Moreover, miR-145 targets p70S6K1 and downregulates HIF-1 and VEGF expression thereby inhibiting tumor growth and angiogenesis [103]. These findings support miR-145 as an important mediator in tumorigenesis and indicate the potential development of the miR-145-based targeted approach for the treatment of colorectal cancer.

## MICRORNAS AS PROMISING THERAPEUTIC TARGETS FOR COLORECTAL CANCER

Exploring the underlying mechanisms that regulate gene expression and the complex signaling pathways is essential in developing novel therapeutics in colorectal cancer treatment. The distinctive ability of microRNAs to target multiple genes and signaling pathways has drawn great attention to their roles as potential innovative therapeutic agents. Generally, microRNA-based therapies include the restoration of downregulated/tumor suppressor microRNA expression or the inhibition of overexpressed/oncogenic microRNAs [110]. For example, the silencing of miR-135b, in which its upregulation corresponds to colorectal tumor progression, reduces the number and size of tumors in a mouse model with no obvious signs of toxicity [111]. In a mouse model of colon carcinoma, intact tumor suppressor miR-145 molecules that are successfully delivered into xenograft tumors result in profound anti-tumor effects by increasing apoptosis and reducing tumor size [98]. Despite functioning as oncogenes or tumor suppressors, microRNAs are also involved in immune responses and their aberrant expressions in the immune system are observed in cancers. Indeed, microRNA-related immunotherapy has recently been employed in colorectal cancer treatment.

More than half of the patients with colorectal cancer experience recurrence and metastasis after surgical resection. Sadly, conventional non-surgical treatment such as chemotherapy is ineffective against metastasis, and the poor prognosis further indicates the urge of developing novel therapeutics that improve clinical outcomes [112]. The immunomodulatory role of microRNAs is gradually recognized and investigated by multiple studies. MicroRNAs have been shown to mediate tumor immune escape by indirectly suppressing T cell activation and proliferation. For example, upregulated MiR-21 and miR-130b in advanced colorectal cancer can inhibit phosphatase and tensin homolog (*PTEN*; a tumor suppressor gene) expression, leading to programmed death ligand 1 (PD-L1) overexpression and immune evasion of colorectal cancer [112, 113]. MiR-124 has been shown to post-transcriptionally target signal transducer and activator of transcription 3 (STAT3), which is overly activated in each stage of colorectal cancer development and is associated with tumor-mediated immunosuppression [112, 114]. Accordingly, Zhang *et al*. observed reduced colorectal cancer cell survival and tumor growth *in vitro* and *in vivo*, respectively, when miR-124, a microRNA known to be downregulated in colorectal cancer, was re-introduced [114]. It is plausible to control inflammatory signaling pathways and immune responses *via* microRNA therapy, thereby decreasing the risk of colorectal tumorigenesis.

The development of microRNA-based treatments, including immunotherapy, remains challenging [110, 112, 115]. Although one single microRNA can regulate a broad set of genes simultaneously, allowing for effective targeting of heterogeneous cancer cells, it is likely to induce off-target side effects due to lack of specificity. The safe delivery and retention of exogenous microRNAs *in vivo* also pose difficulty in microRNA-based treatments [110, 115]. To date, no therapeutic manipulation of microRNAs has been applied in human patients with colorectal cancer; research is only at an experimental stage in either cell lines or animal models. Given the great significance of microRNAs as biomarkers that can be manipulated to reverse tumor progression, we cannot help speculating that a new therapeutic concept--a microRNA-based therapy--might be used in colorectal cancer in the near future. However, standardized microRNA measurement and analysis should be utilized to obtain valid prognostic and diagnostic microRNA profiles in colorectal cancer before their application in the clinical setting.

## CONCLUSIONS

Accumulating evidence has significantly expanded our understanding of microRNA in the pathogenesis of colorectal cancer in all critical progressions of tumorigenesis including cell proliferation, apoptosis, metastasis, and chemoresistance. However, we do not yet completely understand the biology of microRNAs in colorectal cancer development, as well as its association with oxidative stress. In addition, varied methods of handing and processing of microRNA yield inconsistent or incomparable results. It is therefore necessary to establish standardized microRNA protocols and analysis to obtain reliable data and accurately quantify microRNAs by developing a set of stable reference genes for each type of cancer. Besides all the unknowns and difficulties, microRNAs have demonstrated their potential as diagnostic biomarkers. Along with redox therapies targeting cancer cells, the development of personalized therapeutic targets utilizing microRNA for colorectal cancer is feasible. However, the aforementioned challenges should be addressed for the successful transition of microRNAs as prospective biomarkers from bench to clinical setting.

## References

[1] Terzic J, Grivennikov S, Karin E, Karin M (2010). Inflammation and colon cancer. Gastroenterology.

[2] Rex DK, Johnson DA, Anderson JC, Schoenfeld PS, Burke CA, Inadomi JM, American College of G (2009). American College of Gastroenterology guidelines for colorectal cancer screening 2009 [corrected]. Am J Gastroenterol.

[3] Schrader K, Offit K, Stadler ZK (2012). Genetic testing in gastrointestinal cancers: a case-based approach. Oncology (Williston Park).

[4] Haggar FA, Boushey RP (2009). Colorectal cancer epidemiology: incidence, mortality, survival, and risk factors. Clin Colon Rectal Surg.

[5] Huxley RR, Ansary-Moghaddam A, Clifton P, Czernichow S, Parr CL, Woodward M (2009). The impact of dietary and lifestyle risk factors on risk of colorectal cancer: a quantitative overview of the epidemiological evidence. Int J Cancer.

[6] Tuan J, Chen YX (2016). Dietary and Lifestyle Factors Associated with Colorectal Cancer Risk and Interactions with Microbiota: Fiber, Red or Processed Meat and Alcoholic Drinks. Gastrointest Tumors.

[7] Vogelstein B, Fearon ER, Hamilton SR, Kern SE, Preisinger AC, Leppert M, Nakamura Y, White R, Smits AM, Bos JL (1988). Genetic alterations during colorectal-tumor development. N Engl J Med.

[8] O’Connell JB, Maggard MA, Ko CY (2004). Colon cancer survival rates with the new American Joint Committee on Cancer sixth edition staging. J Natl Cancer Inst.

[9] Ambros V (2004). The functions of animal microRNAs. Nature.

[10] Bartel DP (2009). MicroRNAs: target recognition and regulatory functions. Cell.

[11] Kozomara A, Griffiths-Jones S (2011). miRBase: integrating microRNA annotation and deep-sequencing data. Nucleic Acids Res.

[12] Saini HK, Enright AJ, Griffiths-Jones S (2008). Annotation of mammalian primary microRNAs. BMC Genomics.

[13] Liu Z, Wang Y, Borlak J, Tong W (2016). Mechanistically linked serum miRNAs distinguish between drug induced and fatty liver disease of different grades. Sci Rep.

[14] Lee RC, Feinbaum RL, Ambros V (1993). The C. elegans heterochronic gene lin-4 encodes small RNAs with antisense complementarity to lin-14. Cell.

[15] Wightman B, Ha I, Ruvkun G (1993). Posttranscriptional regulation of the heterochronic gene lin-14 by lin-4 mediates temporal pattern formation in C. elegans. Cell.

[16] Kloosterman WP, Plasterk RH (2006). The diverse functions of microRNAs in animal development and disease. Dev Cell.

[17] Lorenzen JM, Haller H, Thum T (2011). MicroRNAs as mediators and therapeutic targets in chronic kidney disease. Nat Rev Nephrol.

[18] Kerr TA, Korenblat KM, Davidson NO (2011). MicroRNAs and liver disease. Transl Res.

[19] Esteller M (2011). Non-coding RNAs in human disease. Nat Rev Genet.

[20] Calin GA, Croce CM (2006). MicroRNA signatures in human cancers. Nat Rev Cancer.

[21] Lu J, Getz G, Miska EA, Alvarez-Saavedra E, Lamb J, Peck D, Sweet-Cordero A, Ebert BL, Mak RH, Ferrando AA, Downing JR, Jacks T, Horvitz HR (2005). MicroRNA expression profiles classify human cancers. Nature.

[22] Aryani A, Denecke B (2015). In vitro application of ribonucleases: comparison of the effects on mRNA and miRNA stability. BMC Res Notes.

[23] Luo X, Stock C, Burwinkel B, Brenner H (2013). Identification and evaluation of plasma microRNAs for early detection of colorectal cancer. PLoS One.

[24] Lan H, Lu H, Wang X, Jin H (2015). MicroRNAs as potential biomarkers in cancer: opportunities and challenges. Biomed Res Int.

[25] Sreevalsan S, Safe S (2013). Reactive Oxygen Species and Colorectal Cancer. Curr Colorectal Cancer Rep.

[26] Tong L, Chuang CC, Wu S, Zuo L (2015). Reactive oxygen species in redox cancer therapy. Cancer Lett.

[27] Perse M (2013). Oxidative stress in the pathogenesis of colorectal cancer: cause or consequence?. Biomed Res Int.

[28] He J, Jiang BH (2016). Interplay between Reactive oxygen Species and MicroRNAs in Cancer. Curr Pharmacol Rep.

[29] Schee K, Boye K, Abrahamsen TW, Fodstad O, Flatmark K (2012). Clinical relevance of microRNA miR-21, miR-31, miR-92a, miR-101, miR-106a and miR-145 in colorectal cancer. BMC Cancer.

[30] Toiyama Y, Takahashi M, Hur K, Nagasaka T, Tanaka K, Inoue Y, Kusunoki M, Boland CR, Goel A (2013). Serum miR-21 as a diagnostic and prognostic biomarker in colorectal cancer. J Natl Cancer Inst.

[31] Burch JA, Soares-Weiser K, DJ St John, Duffy S, Smith S, Kleijnen J, Westwood M (2007). Diagnostic accuracy of faecal occult blood tests used in screening for colorectal cancer: a systematic review. J Med Screen.

[32] Society AC (2011). Colorectal Cancer Facts & Figures 2011-2013.

[33] Zuo L, Zhou T, Pannell BK, Ziegler AC, Best TM (2015). Biological and physiological role of reactive oxygen species—the good, the bad and the ugly. Acta Physiol (Oxf).

[34] He F, Zuo L (2015). Redox Roles of Reactive Oxygen Species in Cardiovascular Diseases. Int J Mol Sci.

[35] Zuo L, Rose BA, Roberts WJ, He F, Banes-Berceli AK (2014). Molecular characterization of reactive oxygen species in systemic and pulmonary hypertension. Am J Hypertens.

[36] Oberreuther-Moschner DL, Rechkemmer G, Pool-Zobel BL (2005). Basal colon crypt cells are more sensitive than surface cells toward hydrogen peroxide, a factor of oxidative stress. Toxicol Lett.

[37] Tagscherer KE, Fassl A, Sinkovic T, Richter J, Schecher S, Macher-Goeppinger S, Roth W (2016). MicroRNA-210 induces apoptosis in colorectal cancer via induction of reactive oxygen. Cancer Cell Int.

[38] Chen Z, Li Y, Zhang H, Huang P, Luthra R (2010). Hypoxia-regulated microRNA-210 modulates mitochondrial function and decreases ISCU and COX10 expression. Oncogene.

[39] Chan SY, Zhang YY, Hemann C, Mahoney CE, Zweier JL, Loscalzo J (2009). MicroRNA-210 controls mitochondrial metabolism during hypoxia by repressing the iron-sulfur cluster assembly proteins ISCU1/2. Cell Metab.

[40] Melnik BC (2015). MiR-21: an environmental driver of malignant melanoma?. J Transl Med.

[41] Jajoo S, Mukherjea D, Kaur T, Sheehan KE, Sheth S, Borse V, Rybak LP, Ramkumar V (2013). Essential role of NADPH oxidase-dependent reactive oxygen species generation in regulating microRNA-21 expression and function in prostate cancer. Antioxid Redox Signal.

[42] Zhou X, Wang X, Huang Z, Wang J, Zhu W, Shu Y, Liu P (2014). Prognostic value of miR-21 in various cancers: an updating meta-analysis. PLoS One.

[43] Zhang X, Ng WL, Wang P, Tian L, Werner E, Wang H, Doetsch P, Wang Y (2012). MicroRNA-21 modulates the levels of reactive oxygen species by targeting SOD3 and TNFalpha. Cancer Res.

[44] Wu CW, Ng SS, Dong YJ, Ng SC, Leung WW, Lee CW, Wong YN, Chan FK, Yu J, Sung JJ (2012). Detection of miR-92a and miR-21 in stool samples as potential screening biomarkers for colorectal cancer and polyps. Gut.

[45] Kanaan Z, Rai SN, Eichenberger MR, Roberts H, Keskey B, Pan J, Galandiuk S (2012). Plasma miR-21: a potential diagnostic marker of colorectal cancer. Ann Surg.

[46] Shibuya H, Iinuma H, Shimada R, Horiuchi A, Watanabe T (2010). Clinicopathological and prognostic value of microRNA-21 and microRNA-155 in colorectal cancer. Oncology.

[47] Xiong BH, Cheng Y, Ma L, Zhang CQ (2013). MiR-21 regulates biological behavior through the PTEN/PI-3 K/Akt signaling pathway in human colorectal cancer cells. International Journal of Oncology.

[48] Drebber U, Lay M, Wedemeyer I, Vallbohmer D, Bollschweiler E, Brabender J, Monig SP, Holscher AH, Dienes HP, Odenthal M (2011). Altered levels of the onco-microRNA 21 and the tumor-supressor microRNAs 143 and 145 in advanced rectal cancer indicate successful neoadjuvant chemoradiotherapy. Int J Oncol.

[49] Deng J, Lei W, Fu JC, Zhang L, Li JH, Xiong JP (2014). Targeting miR-21 enhances the sensitivity of human colon cancer HT-29 cells to chemoradiotherapy in vitro. Biochem Biophys Res Commun.

[50] Yu Y, Sarkar FH, Majumdar AP (2013). Down-regulation of miR-21 Induces Differentiation of Chemoresistant Colon Cancer Cells and Enhances Susceptibility to Therapeutic Regimens. Transl Oncol.

[51] Feng YH, Wu CL, Shiau AL, Lee JC, Chang JG, Lu PJ, Tung CL, Feng LY, Huang WT, Tsao CJ (2012). MicroRNA-21-mediated regulation of Sprouty2 protein expression enhances the cytotoxic effect of 5-fluorouracil and metformin in colon cancer cells. Int J Mol Med.

[52] Han M, Liu M, Wang Y, Mo Z, Bi X, Liu Z, Fan Y, Chen X, Wu C (2012). Re-expression of miR-21 contributes to migration and invasion by inducing epithelial-mesenchymal transition consistent with cancer stem cell characteristics in MCF-7 cells. Mol Cell Biochem.

[53] Qin Y, Yu Y, Dong H, Bian X, Guo X, Dong S (2012). MicroRNA 21 inhibits left ventricular remodeling in the early phase of rat model with ischemia-reperfusion injury by suppressing cell apoptosis. Int J Med Sci.

[54] Yu Y, Kanwar SS, Patel BB, Oh PS, Nautiyal J, Sarkar FH, Majumdar AP (2012). MicroRNA-21 induces stemness by downregulating transforming growth factor beta receptor 2 (TGFbetaR2) in colon cancer cells. Carcinogenesis.

[55] Zhang J, Xiao Z, Lai D, Sun J, He C, Chu Z, Ye H, Chen S, Wang J (2012). miR-21, miR-17 and miR-19a induced by phosphatase of regenerating liver-3 promote the proliferation and metastasis of colon cancer. Br J Cancer.

[56] Horiuchi A, Iinuma H, Akahane T, Shimada R, Watanabe T (2012). Prognostic significance of PDCD4 expression and association with microRNA-21 in each Dukes’ stage of colorectal cancer patients. Oncol Rep.

[57] Lu Z, Liu M, Stribinskis V, Klinge CM, Ramos KS, Colburn NH, Li Y (2008). MicroRNA-21 promotes cell transformation by targeting the programmed cell death 4 gene. Oncogene.

[58] Liu M, Tang Q, Qiu M, Lang N, Li M, Zheng Y, Bi F (2011). miR-21 targets the tumor suppressor RhoB and regulates proliferation, invasion and apoptosis in colorectal cancer cells. FEBS Lett.

[59] Gong B, Liu WW, Nie WJ, Li DF, Xie ZJ, Liu C, Liu YH, Mei P, Li ZJ (2015). MiR-21/RASA1 axis affects malignancy of colon cancer cells via RAS pathways. World J Gastroenterol.

[60] Xiong B, Cheng Y, Ma L, Zhang C (2013). MiR-21 regulates biological behavior through the PTEN/PI-3 K/Akt signaling pathway in human colorectal cancer cells. Int J Oncol.

[61] Chang KH, Miller N, Kheirelseid EA, Ingoldsby H, Hennessy E, Curran CE, Curran S, Smith MJ, Regan M, McAnena OJ, Kerin MJ (2011). MicroRNA-21 and PDCD4 expression in colorectal cancer. Eur J Surg Oncol.

[62] Ma X, Kumar M, Choudhury SN, LE Becker Buscaglia, Barker JR, Kanakamedala K, Liu MF, Li Y (2011). Loss of the miR-21 allele elevates the expression of its target genes and reduces tumorigenesis. Proc Natl Acad Sci U S A.

[63] Xiong Q, Zhong Q, Zhang J, Yang M, Li C, Zheng P, Bi LJ, Ge F (2012). Identification of novel miR-21 target proteins in multiple myeloma cells by quantitative proteomics. J Proteome Res.

[64] Slaby O, Svoboda M, Fabian P, Smerdova T, Knoflickova D, Bednarikova M, Nenutil R, Vyzula R (2007). Altered expression of miR-21, miR-31, miR-143 and miR-145 is related to clinicopathologic features of colorectal cancer. Oncology.

[65] Xu XM, Qian JC, Deng ZL, Cai Z, Tang T, Wang P, Zhang KH, Cai JP (2012). Expression of miR-21, miR-31, miR-96 and miR-135b is correlated with the clinical parameters of colorectal cancer. Oncol Lett.

[66] Motoyama K, Inoue H, Takatsuno Y, Tanaka F, Mimori K, Uetake H, Sugihara K, Mori M (2009). Over- and under-expressed microRNAs in human colorectal cancer. Int J Oncol.

[67] Earle JS, Luthra R, Romans A, Abraham R, Ensor J, Yao H, Hamilton SR (2010). Association of microRNA expression with microsatellite instability status in colorectal adenocarcinoma. J Mol Diagn.

[68] Wang CJ, Zhou ZG, Wang L, Yang L, Zhou B, Gu J, Chen HY, Sun XF (2009). Clinicopathological significance of microRNA-31, -143 and -145 expression in colorectal cancer. Dis Markers.

[69] Chen X, Guo X, Zhang H, Xiang Y, Chen J, Yin Y, Cai X, Wang K, Wang G, Ba Y, Zhu L, Wang J, Yang R (2009). Role of miR-143 targeting KRAS in colorectal tumorigenesis. Oncogene.

[70] Sarver AL, French AJ, Borralho PM, Thayanithy V, Oberg AL, Silverstein KA, Morlan BW, Riska SM, Boardman LA, Cunningham JM, Subramanian S, Wang L, Smyrk TC (2009). Human colon cancer profiles show differential microRNA expression depending on mismatch repair status and are characteristic of undifferentiated proliferative states. BMC Cancer.

[71] Chang KH, Miller N, Kheirelseid EA, Lemetre C, Ball GR, Smith MJ, Regan M, McAnena OJ, Kerin MJ (2011). MicroRNA signature analysis in colorectal cancer: identification of expression profiles in stage II tumors associated with aggressive disease. Int J Colorectal Dis.

[72] Cekaite L, Rantala JK, Bruun J, Guriby M, Agesen TH, Danielsen SA, Lind GE, Nesbakken A, Kallioniemi O, Lothe RA, Skotheim RI (2012). MiR-9,-31, and-182 Deregulation Promote Proliferation and Tumor Cell Survival in Colon Cancer. Neoplasia.

[73] Sun D, Yu F, Ma Y, Zhao R, Chen X, Zhu J, Zhang CY, Chen J, Zhang J (2013). MicroRNA-31 activates the RAS pathway and functions as an oncogenic MicroRNA in human colorectal cancer by repressing RAS p21 GTPase activating protein 1 (RASA1). J Biol Chem.

[74] Nosho K, Igarashi H, Nojima M, Ito M, Maruyama R, Yoshii S, Naito T, Sukawa Y, Mikami M, Sumioka W, Yamamoto E, Kurokawa S, Adachi Y (2014). Association of microRNA-31 with BRAF mutation, colorectal cancer survival and serrated pathway. Carcinogenesis.

[75] Volinia S, Calin GA, Liu CG, Ambs S, Cimmino A, Petrocca F, Visone R, Iorio M, Roldo C, Ferracin M, Prueitt RL, Yanaihara N, Lanza G (2006). A microRNA expression signature of human solid tumors defines cancer gene targets. Proc Natl Acad Sci U S A.

[76] Schetter AJ, Leung SY, Sohn JJ, Zanetti KA, Bowman ED, Yanaihara N, Yuen ST, Chan TL, Kwong DL, Au GK, Liu CG, Calin GA, Croce CM (2008). MicroRNA expression profiles associated with prognosis and therapeutic outcome in colon adenocarcinoma. JAMA.

[77] Monzo M, Navarro A, Bandres E, Artells R, Moreno I, Gel B, Ibeas R, Moreno J, Martinez F, Diaz T, Martinez A, Balague O, Garcia-Foncillas J (2008). Overlapping expression of microRNAs in human embryonic colon and colorectal cancer. Cell Res.

[78] Arndt GM, Dossey L, Cullen LM, Lai A, Druker R, Eisbacher M, Zhang C, Tran N, Fan H, Retzlaff K, Bittner A, Raponi M (2009). Characterization of global microRNA expression reveals oncogenic potential of miR-145 in metastatic colorectal cancer. BMC Cancer.

[79] Bovell LC, Shanmugam C, Putcha BD, Katkoori VR, Zhang B, Bae S, Singh KP, Grizzle WE, Manne U (2013). The prognostic value of microRNAs varies with patient race/ethnicity and stage of colorectal cancer. Clin Cancer Res.

[80] Feng B, Dong TT, Wang LL, Zhou HM, Zhao HC, Dong F, Zheng MH (2012). Colorectal cancer migration and invasion initiated by microRNA-106a. PLoS One.

[81] T Catela Ivkovic, Aralica G, Cacev T, Loncar B, Kapitanovic S (2013). miR-106a overexpression and pRB downregulation in sporadic colorectal cancer. Exp Mol Pathol.

[82] Mosakhani N, Sarhadi VK, Borze I, Karjalainen-Lindsberg ML, Sundstrom J, Ristamaki R, Osterlund P, Knuutila S (2012). MicroRNA profiling differentiates colorectal cancer according to KRAS status. Genes Chromosomes Cancer.

[83] Faltejskova P, Svoboda M, Srutova K, Mlcochova J, Besse A, Nekvindova J, Radova L, Fabian P, Slaba K, Kiss I, Vyzula R, Slaby O (2012). Identification and functional screening of microRNAs highly deregulated in colorectal cancer. J Cell Mol Med.

[84] Kulda V, Pesta M, Topolcan O, Liska V, Treska V, Sutnar A, Rupert K, Ludvikova M, Babuska V, Holubec L, Cerny R (2010). Relevance of miR-21 and miR-143 expression in tissue samples of colorectal carcinoma and its liver metastases. Cancer Genet Cytogenet.

[85] Hofsli E, Sjursen W, Prestvik WS, Johansen J, Rye M, Trano G, Wasmuth HH, Hatlevoll I, Thommesen L (2013). Identification of serum microRNA profiles in colon cancer. Br J Cancer.

[86] Ahmed FE, Jeffries CD, Vos PW, Flake G, Nuovo GJ, Sinar DR, Naziri W, Marcuard SP (2009). Diagnostic microRNA markers for screening sporadic human colon cancer and active ulcerative colitis in stool and tissue. Cancer Genomics Proteomics.

[87] Li JM, Zhao RH, Li ST, Xie CX, Jiang HH, Ding WJ, Du P, Chen W, Yang M, Cui L (2012). Down-regulation of fecal miR-143 and miR-145 as potential markers for colorectal cancer. Saudi Med J.

[88] Kahlert C, Klupp F, Brand K, Lasitschka F, Diederichs S, Kirchberg J, Rahbari N, Dutta S, Bork U, Fritzmann J, Reissfelder C, Koch M, Weitz J (2011). Invasion front-specific expression and prognostic significance of microRNA in colorectal liver metastases. Cancer Sci.

[89] Qian X, Yu J, Yin Y, He J, Wang L, Li Q, Zhang LQ, Li CY, Shi ZM, Xu Q, Li W, Lai LH, Liu LZ (2013). MicroRNA-143 inhibits tumor growth and angiogenesis and sensitizes chemosensitivity to oxaliplatin in colorectal cancers. Cell Cycle.

[90] Gregersen LH, Jacobsen A, Frankel LB, Wen J, Krogh A, Lund AH (2012). MicroRNA-143 down-regulates Hexokinase 2 in colon cancer cells. BMC Cancer.

[91] Zhang Y, Wang Z, Chen M, Peng L, Wang X, Ma Q, Ma F, Jiang B (2012). MicroRNA-143 targets MACC1 to inhibit cell invasion and migration in colorectal cancer. Mol Cancer.

[92] Ng EK, Tsang WP, Ng SS, Jin HC, Yu J, Li JJ, Rocken C, Ebert MP, Kwok TT, Sung JJ (2009). MicroRNA-143 targets DNA methyltransferases 3A in colorectal cancer. Br J Cancer.

[93] Hamfjord J, Stangeland AM, Hughes T, Skrede ML, Tveit KM, Ikdahl T, Kure EH (2012). Differential expression of miRNAs in colorectal cancer: comparison of paired tumor tissue and adjacent normal mucosa using high-throughput sequencing. PLoS One.

[94] Schepeler T, Reinert JT, Ostenfeld MS, Christensen LL, Silahtaroglu AN, Dyrskjot L, Wiuf C, Sorensen FJ, Kruhoffer M, Laurberg S, Kauppinen S, Orntoft TF, Andersen CL (2008). Diagnostic and prognostic microRNAs in stage II colon cancer. Cancer Res.

[95] Diosdado B, van de Wiel MA, Terhaar Sive Droste JS, Mongera S, Postma C, Meijerink WJ, Carvalho B, Meijer GA (2009). MiR-17-92 cluster is associated with 13q gain and c-myc expression during colorectal adenoma to adenocarcinoma progression. Br J Cancer.

[96] Yin Y, Yan ZP, Lu NN, Xu Q, He J, Qian X, Yu J, Guan X, Jiang BH, Liu LZ (2013). Downregulation of miR-145 associated with cancer progression and VEGF transcriptional activation by targeting N-RAS and IRS1. Biochim Biophys Acta.

[97] Kamatani A, Nakagawa Y, Akao Y, Maruyama N, Nagasaka M, Shibata T, Tahara T, Hirata I (2013). Downregulation of anti-oncomirs miR-143/145 cluster occurs before APC gene aberration in the development of colorectal tumors. Med Mol Morphol.

[98] Ibrahim AF, Weirauch U, Thomas M, Grunweller A, Hartmann RK, Aigner A (2011). MicroRNA replacement therapy for miR-145 and miR-33a is efficacious in a model of colon carcinoma. Cancer Res.

[99] Qin J, Wang F, Jiang H, Xu J, Jiang Y, Wang Z (2015). MicroRNA-145 suppresses cell migration and invasion by targeting paxillin in human colorectal cancer cells. Int J Clin Exp Pathol.

[100] Panza A, Votino C, Gentile A, Valvano MR, Colangelo T, Pancione M, Micale L, Merla G, Andriulli A, Sabatino L, Vinciguerra M, Prattichizzo C, Mazzoccoli G (2014). Peroxisome proliferator-activated receptor gamma-mediated induction of microRNA-145 opposes tumor phenotype in colorectal cancer. Biochim Biophys Acta.

[101] Wang Z, Zhang X, Yang Z, Du H, Wu Z, Gong J, Yan J, Zheng Q (2012). MiR-145 regulates PAK4 via the MAPK pathway and exhibits an antitumor effect in human colon cells. Biochem Biophys Res Commun.

[102] Zhang J, Guo H, Zhang H, Wang H, Qian G, Fan X, Hoffman AR, Hu JF, Ge S (2011). Putative tumor suppressor miR-145 inhibits colon cancer cell growth by targeting oncogene Friend leukemia virus integration 1 gene. Cancer.

[103] Xu Q, Liu LZ, Qian X, Chen Q, Jiang Y, Li D, Lai L, Jiang BH (2012). MiR-145 directly targets p70S6K1 in cancer cells to inhibit tumor growth and angiogenesis. Nucleic Acids Res.

[104] Yamada N, Noguchi S, Mori T, Naoe T, Maruo K, Akao Y (2013). Tumor-suppressive microRNA-145 targets catenin delta-1 to regulate Wnt/beta-catenin signaling in human colon cancer cells. Cancer Lett.

[105] Liu L, Chen L, Xu Y, Li R, Du X (2010). microRNA-195 promotes apoptosis and suppresses tumorigenicity of human colorectal cancer cells. Biochem Biophys Res Commun.

[106] Zhang J, Guo H, Qian G, Ge S, Ji H, Hu X, Chen W (2010). MiR-145, a new regulator of the DNA fragmentation factor-45 (DFF45)-mediated apoptotic network. Mol Cancer.

[107] Cancer Genome Atlas N (2012). Comprehensive molecular characterization of human colon and rectal cancer. Nature.

[108] Bastide P, Darido C, Pannequin J, Kist R, Robine S, Marty-Double C, Bibeau F, Scherer G, Joubert D, Hollande F, Blache P, Jay P (2007). Sox9 regulates cell proliferation and is required for Paneth cell differentiation in the intestinal epithelium. J Cell Biol.

[109] ML Marcker Espersen, Linnemann D, Christensen IJ, Alamili M, Troelsen JT, Hogdall E (2016). SOX9 expression predicts relapse of stage II colon cancer patients. Hum Pathol.

[110] Kong YW, Ferland-McCollough D, Jackson TJ, Bushell M (2012). microRNAs in cancer management. Lancet Oncol.

[111] Valeri N, Braconi C, Gasparini P, Murgia C, Lampis A, Paulus-Hock V, Hart JR, Ueno L, Grivennikov SI, Lovat F, Paone A, Cascione L, Sumani KM (2014). MicroRNA-135b promotes cancer progression by acting as a downstream effector of oncogenic pathways in colon cancer. Cancer Cell.

[112] Li X, Nie J, Mei Q, Han WD (2016). MicroRNAs: Novel immunotherapeutic targets in colorectal carcinoma. World J Gastroenterol.

[113] Zhu J, Chen L, Zou L, Yang P, Wu R, Mao Y, Zhou H, Li R, Wang K, Wang W, Hua D, Zhang X (2014). MiR-20b, -21, and -130b inhibit PTEN expression resulting in B7-H1 over-expression in advanced colorectal cancer. Hum Immunol.

[114] Zhang J, Lu Y, Yue X, Li H, Luo X, Wang Y, Wang K, Wan J (2013). MiR-124 suppresses growth of human colorectal cancer by inhibiting STAT3. PLoS One.

[115] Chen Y, Gao DY, In Huang L (2015). vivo delivery of miRNAs for cancer therapy: challenges and strategies. Adv Drug Deliv Rev.

[116] Bandres E, Cubedo E, Agirre X, Malumbres R, Zarate R, Ramirez N, Abajo A, Navarro A, Moreno I, Monzo M, Garcia-Foncillas J (2006). Identification by Real-time PCR of 13 mature microRNAs differentially expressed in colorectal cancer and non-tumoral tissues. Mol Cancer.

[117] Schmitz KJ, Hey S, Schinwald A, Wohlschlaeger J, Baba HA, Worm K, Schmid KW (2009). Differential expression of microRNA 181b and microRNA 21 in hyperplastic polyps and sessile serrated adenomas of the colon. Virchows Arch.

[118] Chiang Y, Song Y, Wang Z, Chen Y, Yue Z, Xu H, Xing C, Liu Z (2011). Aberrant expression of miR-203 and its clinical significance in gastric and colorectal cancers. J Gastrointest Surg.

[119] Chang KH, Mestdagh P, Vandesompele J, Kerin MJ, Miller N (2010). MicroRNA expression profiling to identify and validate reference genes for relative quantification in colorectal cancer. BMC Cancer.

[120] Slattery ML, Wolff E, Hoffman MD, Pellatt DF, Milash B, Wolff RK (2011). MicroRNAs and colon and rectal cancer: differential expression by tumor location and subtype. Genes Chromosomes Cancer.

[121] Wang YX, Zhang XY, Zhang BF, Yang CQ, Chen XM, Gao HJ (2010). Initial study of microRNA expression profiles of colonic cancer without lymph node metastasis. J Dig Dis.

[122] Nishida N, Nagahara M, Sato T, Mimori K, Sudo T, Tanaka F, Shibata K, Ishii H, Sugihara K, Doki Y, Mori M (2012). Microarray analysis of colorectal cancer stromal tissue reveals upregulation of two oncogenic miRNA clusters. Clin Cancer Res.

[123] Wu CW, Dong YJ, Liang QY, He XQ, Ng SSM, Chan FKL, Sung JJY, Yu J (2013). MicroRNA-18a Attenuates DNA Damage Repair through Suppressing the Expression of Ataxia Telangiectasia Mutated in Colorectal Cancer. Plos One.

[124] Nakajima G, Hayashi K, Xi Y, Kudo K, Uchida K, Takasaki K, Yamamoto M, Ju J (2006). Non-coding MicroRNAs hsa-let-7g and hsa-miR-181b are Associated with Chemoresponse to S-1 in Colon Cancer. Cancer Genomics Proteomics.

[125] Xi Y, Formentini A, Chien M, Weir DB, Russo JJ, Ju J, Kornmann M, Ju J (2006). Prognostic Values of microRNAs in Colorectal Cancer. Biomark Insights.

[126] Luo H, Zou J, Dong Z, Zeng Q, Wu D, Liu L (2012). Up-regulated miR-17 promotes cell proliferation, tumour growth and cell cycle progression by targeting the RND3 tumour suppressor gene in colorectal carcinoma. Biochem J.

[127] Sun K, Wang W, Zeng JJ, Wu CT, Lei ST, Li GX (2011). MicroRNA-221 inhibits CDKN1C/p57 expression in human colorectal carcinoma. Acta Pharmacol Sin.

[128] Balaguer F, Moreira L, Lozano JJ, Link A, Ramirez G, Shen Y, Cuatrecasas M, Arnold M, Meltzer SJ, Syngal S, Stoffel E, Jover R, Llor X (2011). Colorectal cancers with microsatellite instability display unique miRNA profiles. Clin Cancer Res.

[129] Michael MZ, O’Connor SM, NG van Holst Pellekaan, Young GP, James RJ (2003). Reduced accumulation of specific microRNAs in colorectal neoplasia. Mol Cancer Res.

[130] Akao Y, Nakagawa Y, Naoe T (2006). MicroRNAs 143 and 145 are possible common onco-microRNAs in human cancers. Oncol Rep.

[131] Reid JF, Sokolova V, Zoni E, Lampis A, Pizzamiglio S, Bertan C, Zanutto S, Perrone F, Camerini T, Gallino G, Verderio P, Leo E, Pilotti S (2012). miRNA profiling in colorectal cancer highlights miR-1 involvement in MET-dependent proliferation. Mol Cancer Res.

[132] Wang X, Wang J, Ma H, Zhang J, Zhou X (2012). Downregulation of miR-195 correlates with lymph node metastasis and poor prognosis in colorectal cancer. Med Oncol.

[133] Chiang Y, Song Y, Wang Z, Liu Z, Gao P, Liang J, Zhu J, Xing C, Xu H (2012). microRNA-192, -194 and -215 are frequently downregulated in colorectal cancer. Exp Ther Med.

[134] Dai X, Chiang Y, Wang Z, Song Y, Lu C, Gao P, Xu H (2012). Expression levels of microRNA-375 in colorectal carcinoma. Mol Med Rep.

[135] Ma Q, Wang X, Li Z, Li B, Ma F, Peng L, Zhang Y, Xu A, Jiang B (2013). microRNA-16 represses colorectal cancer cell growth in vitro by regulating the p53/survivin signaling pathway. Oncol Rep.

[136] Akao Y, Nakagawa Y, Naoe T (2006). let-7 microRNA functions as a potential growth suppressor in human colon cancer cells. Biol Pharm Bull.

[137] Fang WJ, Lin CZ, Zhang HH, Qian J, Zhong L, Xu N (2007). Detection of let-7a microRNA by real-time PCR in colorectal cancer: a single-centre experience from China. J Int Med Res.

[138] Ma Y, Zhang P, Wang F, Zhang H, Yang J, Peng J, Liu W, Qin H (2012). miR-150 as a potential biomarker associated with prognosis and therapeutic outcome in colorectal cancer. Gut.

[139] Zhang Y, He X, Liu Y, Ye Y, Zhang H, He P, Zhang Q, Dong L, Liu Y, Dong J (2012). microRNA-320a inhibits tumor invasion by targeting neuropilin 1 and is associated with liver metastasis in colorectal cancer. Oncol Rep.

[140] Nie J, Liu L, Zheng W, Chen L, Wu X, Xu Y, Du X, Han W (2012). microRNA-365, down-regulated in colon cancer, inhibits cell cycle progression and promotes apoptosis of colon cancer cells by probably targeting Cyclin D1 and Bcl-2. Carcinogenesis.

[141] Yamakuchi M, Yagi S, Ito T, Lowenstein CJ (2011). MicroRNA-22 regulates hypoxia signaling in colon cancer cells. PLoS One.

[142] Roy S, Levi E, Majumdar AP, Sarkar FH (2012). Expression of miR-34 is lost in colon cancer which can be re-expressed by a novel agent CDF. J Hematol Oncol.

[143] Li XM, Wang AM, Zhang J, Yi H (2011). Down-regulation of miR-126 expression in colorectal cancer and its clinical significance. Med Oncol.

[144] Tang JT, Wang JL, Du W, Hong J, Zhao SL, Wang YC, Xiong H, Chen HM, Fang JY (2011). MicroRNA 345, a methylation-sensitive microRNA is involved in cell proliferation and invasion in human colorectal cancer. Carcinogenesis.

[145] Liang Y, Zhao Q, Fan L, Zhang Z, Tan B, Liu Y, Li Y (2015). Down-regulation of MicroRNA-381 promotes cell proliferation and invasion in colon cancer through up-regulation of LRH-1. Biomed Pharmacother.

[146] Zhang Y, Lin C, Liao G, Liu S, Ding J, Tang F, Wang Z, Liang X, Li B, Wei Y, Huang Q, Li X, Tang B (2015). MicroRNA-506 suppresses tumor proliferation and metastasis in colon cancer by directly targeting the oncogene EZH2. Oncotarget.

[147] Fang Y, Sun B, Li Z, Chen Z, Xiang J (2015). MiR-622 inhibited colorectal cancer occurrence and metastasis by suppressing K-Ras. Mol Carcinog.

[148] Huang Z, Huang D, Ni S, Peng Z, Sheng W, Du X (2010). Plasma microRNAs are promising novel biomarkers for early detection of colorectal cancer. Int J Cancer.

[149] Wang Q, Huang Z, Ni S, Xiao X, Xu Q, Wang L, Huang D, Tan C, Sheng W, Du X (2012). Plasma miR-601 and miR-760 are novel biomarkers for the early detection of colorectal cancer. PLoS One.

[150] Chen X, Ba Y, Ma L, Cai X, Yin Y, Wang K, Guo J, Zhang Y, Chen J, Guo X, Li Q, Li X, Wang W (2008). Characterization of microRNAs in serum: a novel class of biomarkers for diagnosis of cancer and other diseases. Cell Res.

[151] Pu XX, Huang GL, Guo HQ, Guo CC, Li H, Ye S, Ling S, Jiang L, Tian Y, Lin TY (2010). Circulating miR-221 directly amplified from plasma is a potential diagnostic and prognostic marker of colorectal cancer and is correlated with p53 expression. J Gastroenterol Hepatol.

[152] Wang B, Zhang Q (2012). The expression and clinical significance of circulating microRNA-21 in serum of five solid tumors. J Cancer Res Clin Oncol.

[153] Ng EK, Chong WW, Jin H, Lam EK, Shin VY, Yu J, Poon TC, Ng SS, Sung JJ (2009). Differential expression of microRNAs in plasma of patients with colorectal cancer: a potential marker for colorectal cancer screening. Gut.

[154] Cheng H, Zhang L, Cogdell DE, Zheng H, Schetter AJ, Nykter M, Harris CC, Chen K, Hamilton SR, Zhang W (2011). Circulating plasma MiR-141 is a novel biomarker for metastatic colon cancer and predicts poor prognosis. PLoS One.

[155] Link A, Balaguer F, Shen Y, Nagasaka T, Lozano JJ, Boland CR, Goel A (2010). Fecal MicroRNAs as novel biomarkers for colon cancer screening. Cancer Epidemiol Biomarkers Prev.

[156] Koga Y, Yasunaga M, Takahashi A, Kuroda J, Moriya Y, Akasu T, Fujita S, Yamamoto S, Baba H, Matsumura Y (2010). MicroRNA expression profiling of exfoliated colonocytes isolated from feces for colorectal cancer screening. Cancer Prev Res (Phila).

[157] Koga Y, Yamazaki N, Yamamoto Y, Yamamoto S, Saito N, Kakugawa Y, Otake Y, Matsumoto M, Matsumura Y (2013). Fecal miR-106a is a useful marker for colorectal cancer patients with false-negative results in immunochemical fecal occult blood test. Cancer Epidemiol Biomarkers Prev.

[158] Kalimutho M, Del Vecchio Blanco G, Di Cecilia S, Sileri P, Cretella M, Pallone F, Federici G, Bernardini S (2011). Differential expression of miR-144* as a novel fecal-based diagnostic marker for colorectal cancer. J Gastroenterol.

[159] Lu MH, Huang CC, Pan MR, Chen HH, Hung WC (2012). Prospero homeobox 1 promotes epithelial-mesenchymal transition in colon cancer cells by inhibiting E-cadherin via miR-9. Clin Cancer Res.

[160] Wu CW, Dong YJ, Liang QY, He XQ, Ng SS, Chan FK, Sung JJ, Yu J (2013). MicroRNA-18a attenuates DNA damage repair through suppressing the expression of ataxia telangiectasia mutated in colorectal cancer. PLoS One.

[161] Yu G, Li H, Wang X, Wu T, Zhu J, Huang S, Wan Y, Tang J (2013). MicroRNA-19a targets tissue factor to inhibit colon cancer cells migration and invasion. Mol Cell Biochem.

[162] Benderska N, Dittrich AL, Knaup S, Rau TT, Neufert C, Wach S, Fahlbusch FB, Rauh M, Wirtz RM, Agaimy A, Srinivasan S, Mahadevan V, Rummele P (2015). miRNA-26b Overexpression in Ulcerative Colitis-associated Carcinogenesis. Inflamm Bowel Dis.

[163] Su SF, Chang YW, Andreu-Vieyra C, Fang JY, Yang Z, Han B, Lee AS, Liang G (2013). miR-30d, miR-181a and miR-199a-5p cooperatively suppress the endoplasmic reticulum chaperone and signaling regulator GRP78 in cancer. Oncogene.

[164] Wu W, Yang J, Feng X, Wang H, Ye S, Yang P, Tan W, Wei G, Zhou Y (2013). MicroRNA-32 (miR-32) regulates phosphatase and tensin homologue (PTEN) expression and promotes growth, migration, and invasion in colorectal carcinoma cells. Mol Cancer.

[165] Li C, Wang Y, Lu S, Zhang Z, Meng H, Liang L, Zhang Y, Song B (2015). miR34a inhibits colon cancer proliferation and metastasis by inhibiting plateletderived growth factor receptor alpha. Mol Med Rep.

[166] Li X, Zhao H, Zhou X, Song L (2015). Inhibition of lactate dehydrogenase A by microRNA-34a resensitizes colon cancer cells to 5-fluorouracil. Mol Med Rep.

[167] Mandke P, Wyatt N, Fraser J, Bates B, Berberich SJ, Markey MP (2012). MicroRNA-34a modulates MDM4 expression via a target site in the open reading frame. PLoS One.

[168] Wu J, Wu G, Lv L, Ren YF, Zhang XJ, Xue YF, Li G, Lu X, Sun Z, Tang KF (2012). MicroRNA-34a inhibits migration and invasion of colon cancer cells via targeting to Fra-1. Carcinogenesis.

[169] Yamakuchi M, Ferlito M, Lowenstein CJ (2008). miR-34a repression of SIRT1 regulates apoptosis. Proc Natl Acad Sci U S A.

[170] Zhang G, Zhou H, Xiao H, Liu Z, Tian H, Zhou T (2014). MicroRNA-92a functions as an oncogene in colorectal cancer by targeting PTEN. Dig Dis Sci.

[171] Huang Z, Huang S, Wang Q, Liang L, Ni S, Wang L, Sheng W, He X, Du X (2011). MicroRNA-95 promotes cell proliferation and targets sorting Nexin 1 in human colorectal carcinoma. Cancer Res.

[172] Qin J, Teng JA, Zhu Z, Chen JX, Wu YY (2015). Glargine Promotes Human Colorectal Cancer Cell Proliferation via Upregulation of miR-95. Horm Metab Res.

[173] Zhou W, Li X, Liu F, Xiao Z, He M, Shen S, Liu S (2012). MiR-135a promotes growth and invasion of colorectal cancer via metastasis suppressor 1 in vitro. Acta Biochim Biophys Sin (Shanghai).

[174] Zhang XF, Li KK, Gao L, Li SZ, Chen K, Zhang JB, Wang D, Tu RF, Zhang JX, Tao KX, Wang G, Zhang XD (2015). miR-191 promotes tumorigenesis of human colorectal cancer through targeting C/EBPbeta. Oncotarget.

[175] Chen DL, Wang ZQ, Zeng ZL, Wu WJ, Zhang DS, Luo HY, Wang F, Qiu MZ, Wang DS, Ren C, Wang FH, Chiao LJ, Pelicano H (2014). Identification of microRNA-214 as a negative regulator of colorectal cancer liver metastasis by way of regulation of fibroblast growth factor receptor 1 expression. Hepatology.

[176] Liao WT, Li TT, Wang ZG, Wang SY, He MR, Ye YP, Qi L, Cui YM, Wu P, Jiao HL, Zhang C, Xie YJ, Wang JX (2013). microRNA-224 promotes cell proliferation and tumor growth in human colorectal cancer by repressing PHLPP1 and PHLPP2. Clin Cancer Res.

[177] Olaru AV, Yamanaka S, Vazquez C, Mori Y, Cheng Y, Abraham JM, Bayless TM, Harpaz N, Selaru FM, Meltzer SJ (2013). MicroRNA-224 negatively regulates p21 expression during late neoplastic progression in inflammatory bowel disease. Inflamm Bowel Dis.

[178] Yuan K, Xie K, Fox J, Zeng H, Gao H, Huang C, Wu M (2013). Decreased levels of miR-224 and the passenger strand of miR-221 increase MBD2, suppressing maspin and promoting colorectal tumor growth and metastasis in mice. Gastroenterology.

[179] Zhang GJ, Zhou H, Xiao HX, Li Y, Zhou T (2013). Up-regulation of miR-224 promotes cancer cell proliferation and invasion and predicts relapse of colorectal cancer. Cancer Cell Int.

[180] Liu X, Zhang Z, Sun L, Chai N, Tang S, Jin J, Hu H, Nie Y, Wang X, Wu K, Jin H, Fan D (2011). MicroRNA-499-5p promotes cellular invasion and tumor metastasis in colorectal cancer by targeting FOXO4 and PDCD4. Carcinogenesis.

[181] Tsang WP, Ng EK, Ng SS, Jin H, Yu J, Sung JJ, Kwok TT (2010). Oncofetal H19-derived miR-675 regulates tumor suppressor RB in human colorectal cancer. Carcinogenesis.

[182] Zhang N, Li X, Wu CW, Dong Y, Cai M, Mok MT, Wang H, Chen J, Ng SS, Chen M, Sung JJ, Yu J (2013). microRNA-7 is a novel inhibitor of YY1 contributing to colorectal tumorigenesis. Oncogene.

[183] Liu Q, Fu H, Sun F, Zhang H, Tie Y, Zhu J, Xing R, Sun Z, Zheng X (2008). miR-16 family induces cell cycle arrest by regulating multiple cell cycle genes. Nucleic Acids Res.

[184] Tsuchiya N, Izumiya M, Ogata-Kawata H, Okamoto K, Fujiwara Y, Nakai M, Okabe A, Schetter AJ, Bowman ED, Midorikawa Y, Sugiyama Y, Aburatani H, Harris CC (2011). Tumor suppressor miR-22 determines p53-dependent cellular fate through post-transcriptional regulation of p21. Cancer Res.

[185] Almeida MI, Nicoloso MS, Zeng L, Ivan C, Spizzo R, Gafa R, Xiao L, Zhang X, Vannini I, Fanini F, Fabbri M, Lanza G, Reis RM (2012). Strand-specific miR-28-5p and miR-28-3p have distinct effects in colorectal cancer cells. Gastroenterology.

[186] Thomas M, Lange-Grunweller K, Weirauch U, Gutsch D, Aigner A, Grunweller A, Hartmann RK (2012). The proto-oncogene Pim-1 is a target of miR-33a. Oncogene.

[187] Kim NH, Cha YH, Kang SE, Lee Y, Lee I, Cha SY, Ryu JK, Na JM, Park C, Yoon HG, Park GJ, Yook JI, Kim HS (2013). p53 regulates nuclear GSK-3 levels through miR-34-mediated Axin2 suppression in colorectal cancer cells. Cell Cycle.

[188] Kim NH, Kim HS, Li XY, Lee I, Choi HS, Kang SE, Cha SY, Ryu JK, Yoon D, Fearon ER, Rowe RG, Lee S, Maher CA (2011). A p53/miRNA-34 axis regulates Snail1-dependent cancer cell epithelial-mesenchymal transition. J Cell Biol.

[189] Tang Q, Zou Z, Zou C, Zhang Q, Huang R, Guan X, Li Q, Han Z, Wang D, Wei H, Gao X, Wang X (2015). MicroRNA-93 suppress colorectal cancer development via Wnt/beta-catenin pathway downregulating. Tumour Biol.

[190] Yang IP, Tsai HL, Hou MF, Chen KC, Tsai PC, Huang SW, Chou WW, Wang JY, Juo SH (2012). MicroRNA-93 inhibits tumor growth and early relapse of human colorectal cancer by affecting genes involved in the cell cycle. Carcinogenesis.

[191] Zhou MK, Liu XJ, Zhao ZG, Cheng YM (2015). MicroRNA-100 functions as a tumor suppressor by inhibiting Lgr5 expression in colon cancer cells. Mol Med Rep.

[192] Liu K, Zhao H, Yao H, Lei S, Lei Z, Li T, Qi H (2013). MicroRNA-124 regulates the proliferation of colorectal cancer cells by targeting iASPP. Biomed Res Int.

[193] Guo C, Sah JF, Beard L, Willson JK, Markowitz SD, Guda K (2008). The noncoding RNA, miR-126, suppresses the growth of neoplastic cells by targeting phosphatidylinositol 3-kinase signaling and is frequently lost in colon cancers. Genes Chromosomes Cancer.

[194] Li N, Li X, Huang S, Shen S, Wang X (2013). [miR-126 inhibits colon cancer proliferation and invasion through targeting IRS1, SLC7A5 and TOM1 gene]. Zhong Nan Da Xue Xue Bao Yi Xue Ban.

[195] Li Z, Li N, Wu M, Li X, Luo Z, Wang X (2013). Expression of miR-126 suppresses migration and invasion of colon cancer cells by targeting CXCR4. Mol Cell Biochem.

[196] Saito Y, Liang G, Egger G, Friedman JM, Chuang JC, Coetzee GA, Jones PA (2006). Specific activation of microRNA-127 with downregulation of the proto-oncogene BCL6 by chromatin-modifying drugs in human cancer cells. Cancer Cell.

[197] Xiang KM, Li XR (2014). MiR-133b acts as a tumor suppressor and negatively regulates TBPL1 in colorectal cancer cells. Asian Pac J Cancer Prev.

[198] Guo H, Hu X, Ge S, Qian G, Zhang J (2012). Regulation of RAP1B by miR-139 suppresses human colorectal carcinoma cell proliferation. Int J Biochem Cell Biol.

[199] Shen K, Liang Q, Xu K, Cui D, Jiang L, Yin P, Lu Y, Li Q, Liu J (2012). MiR-139 inhibits invasion and metastasis of colorectal cancer by targeting the type I insulin-like growth factor receptor. Biochem Pharmacol.

[200] Mi L, Chen Y, Zheng X, Li Y, Zhang Q, Mo D, Yang G (2015). MicroRNA-139-5p Suppresses 3T3-L1 Preadipocyte Differentiation Through Notch and IRS1/PI3K/Akt Insulin Signaling Pathways. J Cell Biochem.

[201] Cai S, Chen J, Xi Z, Zhang L, Niu M, Gao Z (2015). MicroRNA-144 inhibits migration and proliferation in rectal cancer by downregulating ROCK-1. Mol Med Rep.

[202] Zhang H, Li Y, Huang Q, Ren X, Hu H, Sheng H, Lai M (2011). MiR-148a promotes apoptosis by targeting Bcl-2 in colorectal cancer. Cell Death Differ.

[203] Song Y, Xu Y, Wang Z, Chen Y, Yue Z, Gao P, Xing C, Xu H (2012). MicroRNA-148b suppresses cell growth by targeting cholecystokinin-2 receptor in colorectal cancer. Int J Cancer.

[204] Abella V, Valladares M, Rodriguez T, Haz M, Blanco M, Tarrio N, Iglesias P, Aparicio LA, Figueroa A (2012). miR-203 regulates cell proliferation through its influence on Hakai expression. PLoS One.

[205] Wu K, He Y, Li G, Peng J (2015). [Expression and proliferative regulation of miR-204 related to mitochondrial transcription factor A in colon cancer]. Zhonghua Wei Chang Wai Ke Za Zhi.

[206] Song B, Wang Y, Titmus MA, Botchkina G, Formentini A, Kornmann M, Ju J (2010). Molecular mechanism of chemoresistance by miR-215 in osteosarcoma and colon cancer cells. Mol Cancer.

[207] Ma MZ, Chu BF, Zhang Y, Weng MZ, Qin YY, Gong W, Quan ZW (2015). Long non-coding RNA CCAT1 promotes gallbladder cancer development via negative modulation of miRNA-218-5p. Cell Death Dis.

[208] Sun JY, Huang Y, Li JP, Zhang X, Wang L, Meng YL, Yan B, Bian YQ, Zhao J, Wang WZ, Yang AG, Zhang R (2012). MicroRNA-320a suppresses human colon cancer cell proliferation by directly targeting beta-catenin. Biochem Biophys Res Commun.

[209] Zhou C, Liu G, Wang L, Lu Y, Yuan L, Zheng L, Chen F, Peng F, Li X (2013). MiR-339-5p regulates the growth, colony formation and metastasis of colorectal cancer cells by targeting PRL-1. PLoS One.

[210] Wang H, Wu J, Meng X, Ying X, Zuo Y, Liu R, Pan Z, Kang T, Huang W (2011). MicroRNA-342 inhibits colorectal cancer cell proliferation and invasion by directly targeting DNA methyltransferase 1. Carcinogenesis.

[211] Christensen LL, Tobiasen H, Holm A, Schepeler T, Ostenfeld MS, Thorsen K, Rasmussen MH, Birkenkamp-Demtroeder K, Sieber OM, Gibbs P, Lubinski J, Lamy P, Cs group (2013). MiRNA-362-3p induces cell cycle arrest through targeting of E2F1, USF2 and PTPN1 and is associated with recurrence of colorectal cancer. Int J Cancer.

[212] Wang Y, Tang Q, Li M, Jiang S, Wang X (2014). MicroRNA-375 inhibits colorectal cancer growth by targeting PIK3CA. Biochem Biophys Res Commun.

[213] Chai J, Wang S, Han D, Dong W, Xie C, Guo H (2015). MicroRNA-455 inhibits proliferation and invasion of colorectal cancer by targeting RAF proto-oncogene serine/threonine-protein kinase. Tumour Biol.

[214] Guo ST, Jiang CC, Wang GP, Li YP, Wang CY, Guo XY, Yang RH, Feng Y, Wang FH, Tseng HY, Thorne RF, Jin L, Zhang XD (2013). MicroRNA-497 targets insulin-like growth factor 1 receptor and has a tumour suppressive role in human colorectal cancer. Oncogene.

[215] Padi SK, Zhang Q, Rustum YM, Morrison C, Guo B (2013). MicroRNA-627 mediates the epigenetic mechanisms of vitamin D to suppress proliferation of human colorectal cancer cells and growth of xenograft tumors in mice. Gastroenterology.

[216] Xu K, Liang X, Cui D, Wu Y, Shi W, Liu J (2013). miR-1915 inhibits Bcl-2 to modulate multidrug resistance by increasing drug-sensitivity in human colorectal carcinoma cells. Mol Carcinog.

[217] Han HB, Gu J, Zuo HJ, Chen ZG, Zhao W, Li M, Ji DB, Lu YY, Zhang ZQ (2012). Let-7c functions as a metastasis suppressor by targeting MMP11 and PBX3 in colorectal cancer. J Pathol.

